# Developing an integrated intervention to address intimate partner violence and psychological distress in Congolese refugee women in Tanzania

**DOI:** 10.1186/s13031-019-0222-0

**Published:** 2019-08-17

**Authors:** M. Claire Greene, Susan Rees, Samuel Likindikoki, Ann G. Bonz, Amy Joscelyne, Debra Kaysen, Reginald D. V. Nixon, Tasiana Njau, Marian T. A. Tankink, Agnes Tiwari, Peter Ventevogel, Jessie K. K. Mbwambo, Wietse A. Tol

**Affiliations:** 1 Department of Mental Health, Johns Hopkins Bloomberg School of Public Health, Baltimore, MD USA; 2 Department of Psychiatry, Columbia University Medical Center & New York State Psychiatric Institute, 40 Haven Avenue, Rm. 171, New York, NY 10005 USA; 3 Psychiatry Research and Teaching Unit, School of Psychiatry, University of New South Wales, Sydney, NSW Australia; 4 Department of Psychiatry, Muhimbili University of Health and Allied Sciences, Dar es Salaam, Tanzania; 5 HIAS, Silver Spring, MD USA; 6 International Rescue Committee, New York, NY USA; 7 Program for Survivors of Torture, Bellevue Hospital/New York University School of Medicine, New York, NY USA; 8 Department of Psychiatry and Behavioral Sciences, University of Washington, Seattle, WA USA; 9 School of Psychology, Flinders University, Adelaide, Australia; 10 Consultant Anthropological Research & Training on Gender, Violence and Health, Amsterdam, the Netherlands; 11 School of Nursing, The University of Hong Kong, Pok Fu Lam, Hong Kong; 12 Public Health Section, United Nations High Commissioner for Refugees, Geneva, Switzerland; 13 Peter C. Alderman Foundation, HealthRight International, New York, NY USA

**Keywords:** Intimate partner violence, Mental health, Psychological distress, Refugees, Tanzania, Democratic Republic of the Congo, Cognitive processing therapy, Advocacy, Empowerment

## Abstract

**Background:**

Multi-sectoral, integrated interventions have long been recommended for addressing mental health and its social determinants (e.g., gender-based violence) in settings of ongoing adversity. We developed an integrated health and protection intervention to reduce psychological distress and intimate partner violence (IPV), and tested its delivery by lay facilitators in a low-resource refugee setting.

**Methods:**

Formative research to develop the intervention consisted of a structured desk review, consultation with experts and local stakeholders (refugee incentive workers, representatives of humanitarian agencies, and clinical experts), and qualitative interviews (40 free list interviews with refugees, 15 key informant interviews). Given existing efforts by humanitarian agencies to prevent gender-based violence in this particular refugee camp, including with (potential) perpetrators, we focused on a complementary effort to develop an integrated intervention with potential to reduce IPV and associated mental health impacts with female IPV survivors. We enrolled Congolese refugee women with elevated psychological distress and past-year histories of IPV (*n* = 60) who received the intervention delivered by trained and supervised lay refugee facilitators. Relevance, feasibility and acceptability of the intervention were evaluated through quantitative and qualitative interviews with participants. We assessed instrument test-retest reliability (*n* = 24), inter-rater reliability (*n* = 5 interviews), internal consistency, and construct validity (*n* = 60).

**Results:**

We designed an 8-session intervention, termed Nguvu (‘strength’), incorporating brief Cognitive Processing Therapy (focused on helping clients obtaining skills to overcome negative thoughts and self-perceptions and gain control over the impact these have on their lives) and Advocacy Counseling (focused on increasing autonomy, empowerment and strengthening linkages to community supports). On average, participants attended two-thirds of the sessions. In qualitative interviews, participants recommended adaptations to specific intervention components and provided recommendations regarding coordination, retention, safety concerns and intervention participation incentives. Analysis of the performance of outcome instruments overall revealed acceptable reliability and validity.

**Conclusions:**

We found it feasible to develop and implement an integrated, multi-sectoral mental health and IPV intervention in a refugee camp setting. Implementation challenges were identified and may be informative for future implementation and evaluation of multi-sectoral strategies for populations facing ongoing adversity.

**Trial registration:**

ISRCTN65771265, June 27, 2016.

## Background

Refugees may experience a range of adversities, occurring in the period preceding displacement, during displacement, and in the post-migration environment [1, 2]. Stressors can include both past and present potentially traumatic events (PTEs) (e.g., gender-based violence perpetrated by fighting forces; abduction; torture; disappearance of family members) as well as ongoing stressors (e.g., poverty, intimate partner and other forms of violence in refugee camps, lack of access to health care, post-migration living difficulties) [2–5].

Although many individuals display remarkable resilience, a sizeable proportion of refugees experience psychological distress that impairs daily functioning, for example symptoms associated with disorders related to stress, mood, anxiety, and somatoform disorders [6, 7]. When mental health problems occur at a time when refugees are confronted with ongoing social adversity, mental health concerns are typically associated with a worse prognosis for a variety of health and social outcomes [8–12]. Cultural norms and gender inequality may exacerbate the impact that these stressors have on psychosocial wellbeing, interpersonal relationships and violence, and coping strategies. This becomes particularly apparent in traditional patriarchal societies where conflict-related traumatic events and displacement may precipitate rapidly changing gender roles through financial instability, conflict-related violence, loss of identity, and other mechanisms [13].

Social adversity and mental health may be linked in vicious cycles. Epidemiologic evidence suggests that challenges in meeting basic needs, protection, livelihoods, and limited access to education and other social services are related to psychological distress in refugee populations and are both risk factors and consequences of mental health concerns [14–16]. Intimate partner violence (IPV) is a well-documented risk factor for mental health difficulties, including post-traumatic stress, depression and anxiety symptoms [17, 18]. Moreover, mental health concerns as a result of IPV (or other forms of gender-based violence) may in turn be a risk factor for (further) IPV, for example, because self-blame or low self-esteem as a result of violence victimization makes it more challenging to act from a position of strength, especially in contexts of systematic gender-based inequities.

### Intimate partner violence and refugee mental health

The prevalence of IPV in refugees is high, much higher than rates of sexual violence perpetrated outside of the household [19], and is associated with increased risk of mental health sequelae in refugee settings where women often lack options and resources to address these problems and improve their safety [5]. Longitudinal studies have consistently found an association between IPV and the occurrence of depressive symptoms and suicide attempts for women [20–22]. Population-based studies examining the temporality of this relationship have found the onset of mental disorder closely follows experiences of gender-based violence across the lifespan [23]. Moreover, systematic reviews suggest that depressive symptoms are associated with the incidence of IPV in women [20]. For example, longitudinal studies have found that symptoms of depression and post-traumatic stress disorder are associated with presence, severity and intensity of IPV at later time points [22, 24, 25]. Treatment of post-traumatic stress disorder (PTSD) and depression has also been found to reduce rates of IPV revictimization in non-refugees [26]. Treatment of depression in low- and middle-income countries has shown some promising short-term benefits in reducing IPV, but too few mental health treatment studies have been conducted to understand potential impacts in such settings [27].

Although future research is critical to detail the exact mechanisms, the current literature suggests that symptoms of common mental disorder may confer risk for IPV (re-)victimization. Addressing psychological distress may thus be one of multiple important targets for the reduction of IPV, particularly for women in refugee camps and other low resource settings with sufficient resources for women to protect against re-victimization. There is limited empirical research characterizing the exact mechanism underlying the bidirectional association between mental health and IPV [28]. Hypotheses regarding the mechanisms underlying this relationship include the role of mental health problems in partner selection, maintaining abusive relationships or compromising one’s assessment of IPV risk [22]. It is possible that the coercive and controlling behaviors that are characteristics of IPV are employed when women are least equipped to protect themselves, including when women experience psychological distress related to the abuse. Psychological distress, for example ongoing high levels of anxiety, may also make it more difficult for women to deploy safety-planning behaviors that could further protect them from IPV. Conversely, addressing the mental health impacts of IPV may assist in efforts to reduce IPV (re)victimization. Regardless of the exact mechanism by which IPV and mental health problems are related, it is important to support women given the pronounced suffering and functional impairment associated with poor mental health in women affected by IPV [29].

### Development of integrated, multi-sectoral interventions in refugee settings

Previous efforts to integrate survivor-focused IPV prevention and mental health treatment interventions have primarily been implemented and evaluated in high-income countries and have focused largely on addressing IPV and co-occurring substance use and post-traumatic stress symptoms or have largely focused on PTSD and depression [30–37]. These integrated interventions combined elements of safety planning and advocacy, psychoeducation, mindfulness techniques, and cognitive-behavioral therapy delivered through group and/or individual therapy sessions administered by trained healthcare professionals [30–37]. In refugee settings, IPV interventions typically fall within the mandate of protection programming, while mental health services are generally provided by healthcare agencies, with these interventions often provided in isolation. Such unilateral approaches may not address IPV and mental health in ways that lead to lasting change. Humanitarian protection agencies that lack trained personnel, referral options or psychosocial programming may not have the capacity to mitigate the psychological consequences of violence (e.g., reduced self-esteem, hopelessness, self-blame) that may confer risk for further abuse. For mental health agencies, reducing psychological distress in the absence of efforts to prevent IPV, a major risk factor for psychological distress, may undermine the potential for sustained psychological benefits for women who remain at risk for ongoing violence [38, 39]. Therefore, an integrated multi-sectoral strategy that incorporates intervention elements to address both IPV and mental health may operate synergistically and enhance the magnitude and longevity of intervention impacts on both health and protection outcomes [40]. Additionally, integrating health and protection services for IPV survivors may increase the efficiency of service delivery by improving the allocation of trained providers and reducing the need for referrals to other sectors. This approach challenges the assumption that it would be impossible to treat mental health problems and reduce risks relating to IPV if women choose to remain in relationships instead of being relocated to safe spaces or using other physical separation strategies. These approaches to improve mental health and agency of women who have been subjected to IPV need to be complemented with other strategies including primary prevention (e.g., to address the unequal position of women in relationships and in society and to change the norms about acceptability of using violence in interpersonal conflicts) and actions to increase access for IPV survivors to legal, police, housing and financial services [41, 42].

When exploring the potential impacts of integrated IPV and mental health interventions, it is imperative to consider other relevant contributors and correlates of IPV at the family-, community- and socio-cultural levels [43]. Integrated interventions targeting risk factors for IPV, must be aware of unintended consequences, such as victim-blaming, that may arise by focusing on vulnerabilities in IPV-exposed women, rather than on the violent perpetrator and on structural factors that promote violence such as gender inequality. Furthermore, threats to women’s safety in situations where their partners are aware that the services they are receiving are related to IPV and its risk factors must also be carefully monitored and prevented. A multi-sectoral intervention focused on reducing individual-level risk factors should be implemented alongside prevention and protection programming addressing other determinants of IPV (e.g., interventions for male partners, community-based interventions addressing gender norms). Situating an integrated individual-level intervention within a larger multilevel strategy aiming to modify the family-, community- and socio-cultural risk factors may reduce the likelihood of unintentionally placing the burden of responsibility on the survivor and instead recognizing the complex relationships between risk factors and correlates that exist across levels.

### Study objectives

Given that social determinants and mental health are likely interlinked in bi-directional ways, the development of integrated, multi-sectoral interventions that can address both social determinants and mental health at the same time is critical [44]. Although multi-sectoral interventions have been continuously called for since the Alma Ata declaration [45, 46], including in guidance documents relevant to conflict-affected refugees [47], relatively little research has been invested in developing and evaluating integrated, multi-sectoral interventions with refugee populations [48, 49]. Developing, implementing and evaluating multi-sectoral (e.g., protection- and health-sector based) interventions for IPV survivors aligns with the research priorities and recommendations of international experts, health and protection agencies, and funders working in humanitarian settings [50–54].

We aimed to develop a multi-sectoral integrated intervention for psychological distress and IPV, meaning that we introduced a program that combined mental health treatment and IPV prevention intervention components (i.e., integrated) into the protection sector. The multi-sectoral nature of this intervention refers to 1) the inclusion of health priorities, which is usually as seen as outside the scope of protection work, 2) the incorporation of lay personnel trained in both protection and mental health, and 3) coordination with the formal health sector for specific functions such as referral of severe mental health problems and other health concerns. In this paper, we describe formative research aimed at developing a multi-sectoral intervention that integrates a focus on psychological distress and intimate partner violence for refugees in low-resource contexts. More specifically, the objectives of this study were to: (1) conduct formative research to inform the design of a multi-sectoral, integrated survivor-focused mental health treatment and secondary IPV prevention intervention; (2) examine the relevance, acceptability and feasibility of the intervention procedures in a treatment cohort; and (3) prepare and evaluate the outcome assessment tools and research procedures, with Congolese refugee women in Tanzania.

## Methods

### Study setting

This study was conducted in Nyarugusu refugee camp in northwestern Tanzania. At the time the study started, there were over 60,000 Congolese refugees, primarily from the eastern regions of the Democratic Republic of the Congo (DRC) who fled as a result of decades of war [55]. Women and refugees from North and South Kivu in the eastern DRC have experienced widespread gender-based violence [56]. IPV is the most prevalent form of gender-based violence among Congolese refugees living in Nyarugusu camp and continues to be one of the greatest protection priorities [57].

**Fig. 1:**
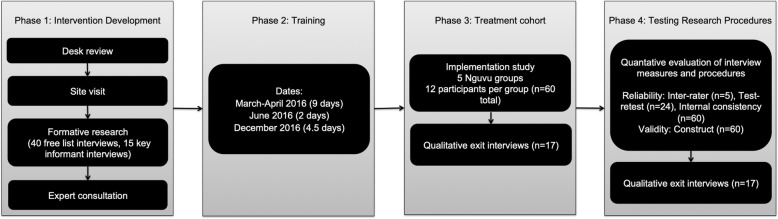
Phases of Nguvu intervention development

The study was implemented in four phases: (1) intervention design; (2) training of facilitators; (3) testing the delivery of the intervention using a treatment cohort; and (4) developing and testing the research tools and procedures. An overview of the flow of research activities is provided in Fig. 1. An overview of the specific indicators and data sources used to evaluate relevance, acceptability, and feasibility of implementation of the intervention and research protocols is provided in Table 1.

**Table 1:** Overview of indicators and data sources used to evaluate implementation of the Nguvu intervention and research protocols

	Intervention Protocol	Research Protocol
Relevance	Indicators: prioritization and burden of problems in Nyarugusu	Indicators: Construct and convergent validity of instruments
	Data sources: Desk review, formative qualitative research	Data sources: Psychometric evaluation of outcome measures
Acceptability	Indicators: Participant retention, perceived benefits and challenges	Indicators: Ethical considerations and safety
	Data sources: Intervention attendance records, qualitative exit interviews	Data sources: Adverse events, qualitative exit interviews
Feasibility	Indicators: Facilitator fidelity and competency	Indicators: Reliability of instruments
	Data sources: Training and supervision notes and feedback	Data sources: Psychometric evaluation of outcome measures

### Phase 1: intervention design

#### Procedures

The intervention was designed by integrating information on the needs of the target population and the relevance of potential intervention components produced by: (1) a desk review; (2) study site visit; (3) formative qualitative research; and (4) expert consultation. The desk review followed methods recommended for humanitarian settings [58, 59] and aimed to summarize existing empirical studies (qualitative and quantitative) and reports on mental health in the context of gender-based violence among eastern Congolese women, with a focus on Congolese refugees in Nyarugusu. We searched for relevant literature in four academic databases (Cochrane Database of Systematic Reviews, Pubmed, PsycINFO and PILOTS) as well as websites and databases with unpublished literature (ReliefWeb, MHPSS.net, Alnap, Refworld, and other sources) using search terms pertaining to the population of interest, general context, humanitarian context, mental health and gender-based violence. We also included articles recommended by experts [60]. In conjunction with the desk review of existing literature, two team members (WT, SL) conducted an initial site visit in Nyarugusu refugee camp to engage with local stakeholders and discuss feasibility of implementing an integrated intervention in this setting in partnership with relevant existing agencies (United Nations High Commissioner for Refugees [UNHCR], Tanzania Red Cross Society, International Rescue Committee).

Following the desk review and preliminary site visit, we conducted a rapid qualitative study with local refugees who were working with humanitarian agencies (i.e., refugee incentive workers) working on projects relating to mental health and/or gender-based violence in Nyarugusu refugee camp. This formative research included 40 free list and 15 in-depth key informant interviews with refugee incentive workers. The objective was to assess their perspective regarding the most common psychological problems experienced by refugees, which groups were most affected by these problems, and what types of services and supports were available. After the free list interviews were completed, the problems were ranked with regard to frequency and saliency. In the key informant interviews, specific attention was given to the perceived causes, symptoms and appropriate strategies to address the three most salient problems.

We formed a team of international and national experts in the fields of refugee mental health and violence protection, to evaluate and select existing evidence-based intervention components based on the results of the desk review, prior systematic reviews [61, 62], the site visit and formative research. We prioritized intervention components that had been previously tested and found to be effective in similar populations or settings (e.g., Congolese women affected by gender-based violence). Intervention components were selected in consultation with these local and international stakeholders based on their relevance, acceptability, and feasibility for implementation in the local refugee context. The expert team was regularly consulted on developing the intervention manual. Tanzanian personnel with training in mental health completed the initial translation of the manual into Swahili, which was revised as needed throughout the training process by facilitators who were familiar with the local dialect.

### Phase 2: training intervention facilitators

#### Participants and sampling

To enhance feasibility and sustainability of the intervention, we followed a task-shifting model [63, 64]. In collaboration with our implementing partners, we identified ten local refugee incentive workers who spoke the local Swahili dialect and had experience with gender-based violence programs in Nyarugusu refugee camp to be trained as intervention facilitators. Trainers consisted of a clinical psychologist with expertise in trauma-informed psychological interventions (AJ) and a medical anthropologist with expertise in community psychiatric nursing and gender-based violence (MT).

#### Procedures

Intervention trainers (AJ, MT) delivered 9 days of training to lay facilitators, which combined training in basic counseling skills, review of the intervention manual consisting of elements of Cognitive Processing Therapy and Advocacy counseling, practice-based role plays and feedback, and self-care strategies (e.g., developing personal self-protection plans). Training primarily occurred through brief didactic sessions in English and facilitated by a Swahili translator with clinical experience followed by role-plays to practice delivering the intervention and receive feedback from trainers and peers. This preliminary training (March–April, 2016, 9 days) was supplemented by two refresher trainings, which were led by mental health professionals (JKKM, SL, TN) in Swahili (June 2016, 2 days; December 2016, 4.5 days). Supervision of sessions provided to participants enrolled in the treatment cohort (see Phase 3) was conducted by phone and intermittent field visits by a clinical supervisor (TN). The clinical supervisor observed sessions and provided feedback to facilitators through group supervision meetings. Facilitators also discussed challenges they were experiencing during these meetings with the clinical supervisor and solutions were discussed amongst the group. Furthermore, if facilitators identified challenging areas of the intervention manual that were difficult for them to deliver or for participants to understand, these were noted, discussed with the supervisor and brought to study investigators for discussion regarding whether adaptations should be made to the manual. Study investigators encouraged the facilitators to provide potential solutions and recommendations to the challenges they faced in order to inform adaptations to the manual and intervention procedures.

### Phase 3: treatment cohort to test the delivery of the intervention

#### Participants and sampling

To evaluate the implementation of an integrated IPV and mental health intervention, we enrolled a cohort of 60 participants to participate in the intervention. The study sample consisted of adult (18+ years) female Congolese refugees living in Nyarugusu refugee camp who had been in an intimate relationship in the last 12 months and reported past-year physical or sexual IPV and elevated symptoms of psychological distress. We did not place restrictions regarding whether the woman was currently in the relationship and/or living with their most recent partner. Screening of past-year physical or sexual IPV was conducted using the Abuse Assessment Screen (AAS) [65]. Psychological distress was operationalized as reporting an average score of 1.75 or greater on the 25-item Hopkins Symptom Checklist (HSCL-25) [66–68], which measures symptoms of anxiety or depression, or an average score of 1.0 or greater on the 16-item Harvard Trauma Questionnaire (HTQ) [69, 70], which measures symptoms of post-traumatic stress. These cutoffs were based on prior research with Congolese women for whom these average scores were found to indicate significant psychological distress [71]. We excluded women with observable signs of severe psychiatric disorder that would impede participation in sessions or were identified to be at imminent risk of suicide through screening procedures. A suicide risk assessment was conducted to evaluate imminent risk of suicide if participants reported thoughts of ending their life during the past 2 weeks, which was an item included on the mental health assessment tools completed by all participants. Participants were recruited through local women’s groups that were led by refugees in villages within one zone in the camp. A research assistant presented this study to members of the women’s group as a women’s health and wellbeing program to avoid women interested in participating in the study being identified as IPV survivors.

#### Procedures and ethics

All participants provided oral consent for screening and, if eligible, written consent to participate in the baseline interview and Nguvu intervention. The baseline interview was intended to complement the information gathered during screening and included additional measures of demographic characteristics, IPV, functional impairment, trauma history and major life events, social capital, safety planning/behavior, coping strategies and service utilization. All study procedures were reviewed and approved by the Johns Hopkins Institutional Review Board (USA), Muhimbili University of Health and Allied Sciences Institutional Review Board (Tanzania), and the National Institute for Medical Research (Tanzania). Eligible participants who consented to participate and completed the baseline interview were assigned to a Nguvu intervention group. Intervention sessions lasted approximately 2 h each and were scheduled to occur weekly over an eight-week period, which was considered a reasonable duration for this type of intervention in the study setting. After completion of the intervention, a subset of participants representing high attenders (i.e., 6–8 sessions) and low attenders (i.e., 0–3 sessions) were selected to participate in qualitative semi-structured interviews to examine the relevance and acceptability of the Nguvu intervention (*n* = 17).

The exit interviews asked questions about safety, benefits, and challenges of the Nguvu intervention. Participants were asked to respond to questions about the utility of specific components of the Nguvu intervention on 4-point Likert scales ranging from “not helpful” to “extremely helpful”. All other questions were open ended and designed to elicit qualitative responses.

Similar to the recruitment process, we avoided publicly labeling Nguvu as an IPV program to preserve confidentiality and promote safety of women participating in the intervention. Questions about safety were integral to the assessments and remained a focus of the intervention sessions. Safety resources available in the camp were discussed with all participants during the first intervention session. Any adverse events were reported to study investigators and evaluated by a Data and Safety Monitoring Board.

#### Analysis

Descriptive statistics of the age, ethnicity, marital status and family composition were calculated to characterize the overall treatment cohort sample (*n* = 60). The feasibility of implementation was further examined using attendance as a quantitative indicator of retention. Participant responses to exit interview questions were reviewed by two researchers who participated in the training and implementation of the treatment cohort. Responses to open-ended questions were summarized using thematic analysis.

### Phase 4: developing and testing assessment tools and research procedures

#### Participants and sampling

All participants recruited for the treatment cohort were included in our evaluation of outcome assessment tools and research procedures. Similarly, the high and low attenders selected for the qualitative exit interviews also responded to questions regarding research procedures during the interviews.

#### Instruments

The primary and secondary outcomes were psychological distress (primary), IPV (primary) and functional impairment (secondary). In addition to screening for IPV and mental health problems using the AAS, HSCL-25 and HTQ, the baseline interview included the assessment of IPV using an adapted version of the Conflict Tactics Scale [72, 73]; trauma history using the Harvard Trauma Questionnaire [69]; IPV safety planning using the Safety-Promoting Behavior Checklist [74]; and measures developed in the eastern DRC to assess functional impairment, major life events, coping and service use, and social capital [71].

#### Procedures

All treatment cohort participants (*n* = 60) completed a screening and baseline interview. A subset (*n* = 24) of eligible participants repeated the screening and baseline interview approximately 1 week after the initial baseline interview in order to examine test-retest reliability. All interviews were conducted by trained research assistants who were also members of the community, most of whom had prior experience in data collection. During interviewer training, inter-rater reliability was evaluated through all interviewers’ observing and independently recording responses from five interviews.

### Statistical analysis

To assess the relevance of the outcome measures, we evaluated convergent validity using baseline data from all 60 participants included in the treatment cohort. We examined convergent validity between all total and subscale scores for primary and secondary outcomes using Spearman rank order correlations. To assess acceptability of the research procedures we used data from the subset of high and low intervention attenders who participated in the qualitative exit interviews after the intervention had been delivered (*n* = 17). Any themes that emerged relating to the research procedures during the coding and thematic analysis process were examined as potential explanations of acceptability, particularly as they related to safety and ethics. Feasibility of the research procedures was assessed using indicators of instrument reliability, which reported on the ability of our measurement tools and methods to consistently assess our primary outcomes across time, items and interviewers. More specifically, test-retest reliability (*r*) examined the consistency in measurement reported by the same participant (*n* = 24) in interviews scheduled to take place 1 week apart. Inter-rater reliability (measured using the intra-class correlation coefficient, ICC) examined consistency in measurement between interviewers observing the same five interviews. Lastly, internal consistency (Cronbach’s α) examined the homogeneity of items within each scale using data from all 60 participants.

## Results

### Phase 1: intervention design

#### Desk review and site visit

Findings from the desk review [60] and consultations with stakeholders during the site visit consistently reported that gender-based violence, particularly IPV, was prevalent among Congolese women from the eastern DRC, including refugees who were displaced from this region. Symptoms of depression, anxiety and post-traumatic stress were reported to be common among Congolese women who had experienced IPV, but very few interventions have been evaluated to address mental health and IPV in this population [60]. The lack of and need for interventions addressing psychological distress in the context of IPV in Nyarugusu refugee camp was reaffirmed in discussions with representatives from UNHCR, the Tanzania Red Cross, International Rescue Committee, as well as refugee incentive workers. Because of the active implementation of programs aimed at primary prevention of gender-based violence in the camp, including with (potential) perpetrators, we decided to focus our efforts on developing an intervention aimed at addressing IPV and mental health with female survivors.

#### Free listing and key informant interviews

Free listing interviews (*n* = 40) with refugees who had experience working with humanitarian agencies in Nyarugusu reported that the three most salient problems affecting women who had experienced IPV translated to stress (*msongo wa mawazo*, literally too many thoughts), sadness (*huzuni*) and fear (*hofu*). Descriptions of these problems provided in the 15 in-depth key informant interviews were broadly consistent with symptoms of common mental disorders (depression, anxiety, and posttraumatic stress) (see Table 2). In addition to women affected by IPV, key informants reported that these psychological problems also commonly affected married and widowed women. The most frequently reported causes were IPV, abandonment, isolation, divorce, infertility and husbands not fulfilling their responsibilities. Counseling and strengthening social support networks were most frequently recommended for management of all three identified problems.

**Table 2:** Results of free listing and key informant interviews investigating priority problems for women in Nyarugusu

	*Msongo wa mawazo* (Stress)	*Huzuni* (Sadness)	*Hofu* (Fear)
Descriptors	Silence, unhappiness, preferring to be alone	Crying, silence, aggressiveness	Preferring to be alone, worrying, feeling shocked, trembling or worrying when she sees her husband
Commonly Affected Groups	Widows, married women, women affected by IPV, women who have been abandoned by partner	Women affected by IPV, married women, women with infertility	Married women, women affected by IPV, widows, single mothers, women living with her partner
Causes	IPV, abandonment, divorce, when husbands do not fulfill their responsibilities	IPV, abandonment, infertility	IPV, isolation
Management	Counseling, helping her with responsibilities, introduce her to other activities and social groups, comfort her, provide support, direct her to legal organizations	Counseling, introduce her to other activities and social groups, comfort her	Counseling, introduce her to other activities or social groups, comfort her, develop a friendship

#### Selection of components

The preceding desk review, initial site visit, and formative research in our interpretation indicated the need for a mental health intervention for IPV survivors that could target psychological distress broadly. We were interested in identifying an evidence-based intervention that could address psychological distress and combine this with activities focused on reducing risk for IPV more directly.

With regard to selection of a mental health intervention, cognitive processing therapy (CPT) was the only rigorously evaluated psychological intervention tested with adult gender-based violence survivors in the context of armed conflict in a low-resource setting, according to the most recent systematic review at the time of writing [62]. CPT was developed to treat mental health sequelae in survivors of sexual violence [75]. CPT is an evidence-based skills-based intervention that equips individuals with strategies to manage distressing thoughts that lead to psychological distress [76]. More specifically, individuals are taught to challenge trauma-related thoughts, such as self-blame and cognitive distortions, using cognitive restructuring techniques to reduce symptoms of psychological distress [77]. CPT delivered by paraprofessionals was adapted and evaluated in Congolese survivors of gender-based violence in the eastern DRC, a region with some of the highest documented rates of gender-based violence globally [78], and was found to be associated with significant reductions in depression, anxiety, post-traumatic stress and functional impairment [71] – and was therefore a good fit for a broad focus on psychological distress. In addition, secondary analyses of this trial found that the intervention increased structural social capital [79], fitting with the stakeholder-recommended focus on strengthening social support networks.

To enhance feasibility in a low-resource, dynamic refugee setting, we selected a 6-session protocol of the CPT intervention [80, 81] as opposed to the 12-session CPT intervention evaluated in the DRC. Previous evaluations of CPT in a high-income country found that 58% of individuals achieved substantive improvement in PTSD and depression with fewer than 12 sessions [82], suggesting that reducing the length of the intervention to improve feasibility may not compromise the effectiveness of CPT. However, it is important to note that the 6-session protocol was developed for acute trauma response and thus it is unclear whether it may as effective for IPV, which is typically a chronic adverse exposure.

With regard to IPV-focused intervention components, advocacy counseling has shown promising results both to reduce IPV and short-term mental health outcomes [83]. In line with recommendations to evaluate empowerment-based advocacy and cognitively focused clinical interventions with IPV survivors [84], we explored the integration of CPT with advocacy counseling [84]. We selected an advocacy counseling approach previous evaluated in Hong Kong [85], as a World Health Organization review found that this intervention had the strongest evidence of effectiveness for reducing IPV [61].

Advocacy counseling focuses on increasing autonomy, empowerment and strengthening linkages to community services by helping survivors process experiences of IPV and explore potential solutions that are supported by the facilitator through goal setting and safety planning [84–86]. Empowerment of women affected by IPV is traditionally rooted in the feminist model and addresses the social and contextual determinants of IPV, including the gendered role of men in society, with the aim of shifting culpability to the perpetrator and social context [87]. Advocacy interventions are thought to empower women through discussion of possible solutions to their IPV situation and facilitating client-directed goals instead of those that are dictated by the provider [88]. A systematic review of advocacy interventions for women affected by IPV found that these interventions may improve quality of life and reduce physical IPV. Furthermore, brief advocacy interventions provided some indication of improving mental health in the short-term [83].

To emphasize the psychological and social empowerment goals of both components of the intervention, we refer to it as Nguvu, the Swahili term for strength. The structure of Nguvu, is detailed in Table 3. The Nguvu intervention was designed as an 8-session intervention that begins with a single individual session of advocacy counseling followed by six group sessions focused primarily on CPT and a final group session of advocacy counseling. The initial individual session was delivered by one facilitator and focused on psychoeducation surrounding IPV and psychological distress, an assessment of the woman’s IPV situation and development of a safety plan. Next, there were six sessions of modified CPT, most of which were approximately 2 h in length. The modified protocol included the following content and associated homework activities: explanation of thoughts and feelings, exploration of stuck thoughts, the ABCs (becoming aware of the connection between an event, the resulting thought, and how this thought makes the person feel), and challenging maladaptive thoughts relating to the themes of safety, trust, power/control, esteem, and care in relation to oneself and others, and how these concepts relate to one’s safety plan. The intervention also added in relaxation training to session three in order to provide a behavioral coping strategy for managing distress. The final session centered on group review of client’s safety plans and discussion of available services, support and coping strategies. All group sessions were led by facilitator pairs, which allowed co-facilitators to complement one another’s strengths, improve group management, and provided an opportunity for peer supervision and support (Table 3).

**Table 3:** Structure of Nguvu intervention

Session	Topic	Description	Homework Activities
1. Empowerment/ Advocacy (120 min)	Advocacy and safety plan	• Information on IPV • Discussing psychological distress • Danger assessment • Safety plan and emergency plan	Safety plan
2. CPT (120 min)	Intro to CPT in Nguvu	• Introducing group rules and overview of Nguvu sessions • Stuck thoughts • Explanation of thoughts and feelings • Treatment goals	Notice and explore thoughts and distress related to IPV
3. CPT + Relaxation (120 min)	ABCs	• Introduction of ABCs • Exploring stuck points • Group relaxation^a^	Daily practice of ABCs and relaxation task
4. CPT (120 min)	Stuck points and thinking questions	• Changing thoughts and feelings • Thinking questions • Exploring stuck thoughts	Daily practice of ABCs and exploration of stuck thoughts; relaxation task
5. CPT (120 min)	Learning safety and trust	• Introduction to safety and trust • Stuck thoughts related to trust	Daily practice of ABCs, thinking questions and changing thoughts
6. CPT (120 min)	Power, control and self-esteem	• Introduction to power/control issues related to self and others • Challenging questions for control issues • Self-esteem • Caring related to self and others	Daily practice of ABCs, thinking questions and changing thoughts; self-care
7. CPT (60–120 min)	CPT Review	• Discuss the impact of distressing events • Planning for the future	Daily practice of ABCs, thinking questions and changing thoughts; self-care; revise safety plan; relaxation exercise
8. Empowerment/ Advocacy (120 min)	Review of advocacy and safety plan	• Review safety plan • Advocacy • Coping and support methods	–

^a^Group relaxation was added to session 3 and is not part of the traditional CPT manual

### Phase 2: training and ongoing capacity building for lay intervention facilitators

We found that it was feasible to train lay community members to deliver Nguvu, which was similar to findings from studies that have utilized paraprofessionals or research staff to deliver CPT and advocacy counseling, respectively [71, 85]. Throughout the training process, cultural adaptations were made to the manual based on recommendations from the facilitators to improve language, comprehensibility and incorporating locally salient examples relating to gender norms and IPV. This served as an advantage of using local community members as facilitators as they were able to recommend ways to improve the relevance and acceptability of the intervention content prior to initial implementation. A supervision structure was proposed to provide ongoing support to the facilitators. Initially, facilitators met regularly with a clinical supervisor, who also directly observed Nguvu sessions and provided feedback to facilitators. We planned to provide intermittent field supervision supplemented by remote supervision after the initial training period, but due to communication challenges we found that it would be necessary to hire a full time clinical supervisor to remain on-site to provide regular in-person supervision. Additionally, the facilitators recommended electing a lead facilitator to serve as a representative for them. The lead facilitator was responsible for communicating with staff supervisors and assist with logistics and management of the facilitator team.

### Phase 3: implementation of Nguvu intervention procedures

#### Sample characteristics

Of the 102 participants screened, 61 (59.8%) met eligibility criteria of which 60 consented to participate in the baseline interview and intervention. The 41 ineligible participants were excluded because they did not meet criteria for past-year IPV (*n* = 7), elevated psychological distress (*n* = 10), they didn’t report having an intimate partner in the past year (*n* = 4), or they did not meet either of the past-year IPV or elevated psychological distress criteria (*n* = 20). On average, participants were 28.6 years old (SD = 10.4), over half were of Bembe ethnicity (59.7%), married (58.3%), and most participants had children (85.0%; 3.9 children, on average). Twenty percent had been selected and begun the process of resettlement to a country other than Tanzania or the DRC at the time of intervention. The majority of eligible participants (86.7%) reported experiencing both physical and sexual violence perpetrated by an intimate partner during the past year on screening measures. Levels of symptoms of post-traumatic stress, depression and anxiety were substantial, with average symptom scores ranging between a “moderate amount” to “a lot” of symptoms during the past 2 weeks, as per the eligibility criteria.

#### Intervention attendance

Each of the 60 participants was allocated to an intervention group led by a pair of facilitators (k = 5 groups, 12 participants per group). As per the intervention protocol, the first session was delivered individually followed by seven group sessions. On average, participants attended 66% of Nguvu sessions, however, with substantial variability by facilitator pair (52–79%). Only two participants did not attend any sessions and approximately half of the participants attended at least 75% of sessions (i.e., 6+ of 8). The highest proportional attendance was observed for session one (92%) with the lowest attendance observed at session six (57%).

#### Intervention implementation

Seven participants who attended three or fewer sessions and ten participants who attended six or more sessions completed exit interviews after the intervention was complete. Overarching themes of these interviews included perceptions of the intervention, retention issues, safety concerns and suggestions for improving Nguvu. In general, the intervention was thought to be relevant and helpful, particularly discussions about IPV and homework assignments relating to daily practice of the ABCs, thinking questions, changing thoughts, and relaxation skills. The most difficult parts of Nguvu to understand were also the ABCs and changing thoughts. The reasons for absence from sessions included health problems, parenting and childcare, as well as prior engagements (e.g., funerals, school, work/chores), and lack of information due to poor communication. To improve retention the participants suggested that information about session scheduling be provided with more advance notice and also to increase communication between Nguvu staff and participants by providing mobile phones and visiting participants’ homes.

We were particularly interested in experiences of safety given the sensitive nature of IPV and the importance of confidentiality to avoid perpetuating or increasing women’s risk for ongoing violence. When asked about safety, all of the seven high attenders reported feeling safe participating in Nguvu, whereas one of eight low-attenders who responded to questions about safety reported not feeling safe at all and two did not provide a response. One participant reported on safety challenges that one of her acquaintances had experienced stating that “because of her husband, she felt like if [the husband] knew it would be a problem and that would complicate the matter even more, considering the husband’s situation”. Participants suggested that improving communication and organization would also improve safety because women would be aware, in advance, of the exact time and date of sessions. Participants did not report concerns about other community members finding out about their participation - safety concerns primarily were related to their partner’s awareness.

Participants also provided other recommendations for improving Nguvu prior to implementation of a randomized feasibility trial. These suggestions included involving male partners (i.e., husband, boyfriend) and providing services for them, improving communication and organization, providing food and other incentives for attending sessions, increasing the number and continuity of sessions, and incorporating different projects and activities into the intervention.

### Phase 4: implementation of Nguvu research procedures

#### Relevance - convergent validity

Bivariate correlations between primary and secondary outcomes revealed good external construct validity indicated by moderate correlation coefficients, particularly between mental health constructs (Table 4). The psychological distress subscales were significantly correlated with one or more forms of IPV (range: 0.23–0.39) and PTSD symptoms were significantly associated with functional impairment (*r* = 0.31). In general, IPV was most strongly correlated with symptoms of PTSD.

**Table 4:** Spearman correlation between primary and secondary outcomes

	1	2	3	4	5	6	7	8	9	10
1. All Psychological Distress Items										
2. HSCL-25	0.956***									
3. HSCL (Anxiety)	0.833***	0.868***								
4. HSCL (Depression)	0.790***	0.835***	0.482***							
5. HTQ (PTSD)	0.915***	0.781***	0.706***	0.594***						
6. All IPV Items	0.394**	0.333*	0.232	0.313*	0.392**					
7. Psychological IPV	0.388**	0.322*	0.200	0.348**	0.407**	0.835***				
8. Physical IPV	0.311*	0.253	0.166	0.246	0.314*	0.939***	0.654***			
9. Sexual IPV	0.333*	0.332*	0.302*	0.218	0.278*	0.742***	0.683***	0.526***		
10. Functioning (Original)	0.306*	0.245	0.183	0.234	0.346**	0.237	0.285*	0.145	0.278*	
11. Functioning (Reduced)	0.291*	0.239	0.232	0.156	0.317*	0.232	0.279	0.142	0.285*	0.935*

*Abbreviations*: *HSCL* Hopkins symptom checklist, *HSCL-25* 25-item Hopkins symptom checklist 25, *HTQ* Harvard trauma questionnaire, *IPV* Intimate partner violence, *PTSD* Post-traumatic stress disorder

*p < 0.05, **p < 0.01, ***p < 0.001

#### Acceptability - ethical issues and safety

In the qualitative exit interviews participants did not mention safety or ethical concerns relating to the research procedures. Furthermore, we did not identify adverse events related to the research procedures during the treatment cohort. We identified specific adaptations to the allocation procedures that were needed to improve acceptability. For example, during supervision the facilitators described that women preferred that the intervention groups be more homogeneous in terms of age composition. More specifically, women reported having elder relatives as members of their intervention group and they were uncomfortable disclosing information about violence occurring within their relationship to these relatives.

#### Feasibility - reliability of outcome instruments

Examination of the primary outcome measures revealed overall good test-retest reliability, inter-rater reliability and internal consistency (see Table 5). Test-retest reliability of the sexual IPV items was low (*r* = 0.452). Additionally, internal consistency of the functional impairment and psychological IPV items was low (functioning: α = 0.144, psychological IPV: α = 0.623). Inspection of the item-rest correlations revealed several items that were weakly correlated with the remaining items and perhaps unrelated to functioning in the sample such as farming, trading and other income-generating activities. Determining which items to remove was based on low item-rest correlations as well as discussion with local staff about the relevance of given items to the concept of functioning among women in Nyarugusu camp. Removal of ten items on the functional impairment measure improved internal consistency to an acceptable level (α = 0.797).

**Table 5:** Reliability of primary and secondary outcomes

	Number of items	Test-Retest Reliability (r)	Inter-Rater Reliability [ICC (3,1)]	Internal Consistency (α)
Mental Health				
HSCL-25	25	0.729	0.902	0.765
HTQ	16	0.786	0.999	0.762
Intimate Partner Violence (Frequency)				
Psychological	2	0.722	1.000^a^	0.623
Physical	7	0.795	0.980^a^	0.863
Sexual	2	0.452	0.989^a^	0.889
Functional Impairment	22	0.620	0.904^a^	0.144

*HSCL-25* Hopkins-symptom checklist for anxiety and depressive symptoms, *HTQ* Harvard trauma questionnaire for post-traumatic stress symptoms

^a^Average of inter-rater reliability for IPV and functional impairment items because average score not calculated by interviewers

## Discussion

In this article we present the development of a multi-sectoral, integrated intervention to reduce psychological distress and IPV in refugees. This study has important implications for future evaluations of Nguvu as well as the development and adaptation of integrated interventions in refugee populations more broadly. Based on the formative research, which was later supported by our treatment cohort data, we found that an 8-session intervention could be designed that addresses two critical priorities identified both in desk review and local stakeholder consultation. The priority need was for a mental health intervention addressing distress broadly, and a group intervention that can address need for social support strengthening. CPT, which was the only rigorously tested mental health intervention for survivors of gender-based violence in post-conflict settings was a good fit, but needed to be shortened from 12 to 6 sessions to increase feasibility. Training and supervision indicated that delivery of Nguvu by lay refugee workers was feasible, which has critical implications for the scalability of this intervention. Employing an apprenticeship models that allows for local training and supervision of lay facilitators increases the likelihood that Nguvu can be sustainable in humanitarian and low-resource settings [63]. We did not identify any adverse events throughout the course of the study nor other indications that participation in an intervention like Nguvu would increase risk of IPV for women who remained in relationships with their partner. It is also important to note that we closely monitored and made considerable efforts to protect the safety and confidentiality of treatment cohort participants and similar precautions should be taken in future efforts to implement this type of intervention for IPV survivors who remain at risk for ongoing IPV.

In addition to shortening the intervention, we had to adapt several content and procedural aspects of group-based CPT and advocacy counseling. These adaptations included modifying content to improve comprehensibility and relevance. Throughout the formative research and treatment cohort phases we elicited recommendations for future adaptations from a variety of stakeholders. Participants recommended that the age composition of intervention groups be homogenized to avoid having relatives of different generations within the same group, which can result in relational power dynamics that may inhibit group discussion. In the future, we plan to introduce age-specific allocation procedures that will ensure that participants are of similar age to other group members. Re-evaluating and adapting our retention strategies was also a critical lesson learned through this study. The exit interviews highlighted that communication was a challenge and participants recommended that staff visit them in their homes to remind them of session schedules. Improving organization through better engagement with partners and better communication with participants was consistently discussed and something that should be the focus of future efforts to implement integrated programs. Participants also recommended providing incentives and introducing different projects or activities into the intervention to improve Nguvu. Providing incentives for delivering services that are intended to improve wellbeing may compromise the scientific integrity of evaluations, preclude exploration of the mechanisms behind changes in psychological distress or violence, and diminish changes for sustainability of the intervention. On the other hand, the integration of livelihood activities may serve to tackle other critical risk factors, such as poverty, that may similarly relate to both psychological distress and IPV. Another recommendation was to increase the number of sessions and continuity of Nguvu in Nyarugusu. This was promising feedback as it suggested that the intervention was perceived as helpful for the community and they suggested expanding the reach of the intervention. In addition to increasing the number of sessions, it is possible that modifying the relative dose of the empowerment and advocacy content to the number of CPT sessions may improve and optimize outcomes.

We often received feedback suggesting that male partners should be somehow involved in order to prevent the perpetration of violence. Nguvu was designed to be delivered in settings where other prevention activities are occurring, including efforts to reduce perpetration of violence targeting men. Nyarugusu was selected as the study site, in part because there have been several initiatives focused on addressing gender norms and risk factors for violence among men. Community-based interventions intended to promote positive masculinity, gender equality and develop non-violence, adaptive coping strategies for men have been implemented and evaluated in low-resource settings including among Congolese men in the eastern DRC [89]. Although qualitative and quantitative evaluations of community-based interventions targeting masculinity and gender norms have found some promising results related to family and community violence, the evidence-base surrounding these interventions remains mixed and should be the focus of future research [89–91], including potential integration of services for men into a program like Nguvu. One concern, however, is how best to integrate services for survivors with services for men and community-based programs without compromising the protection and safety of women. Evaluations of couples interventions that incorporate elements of CPT and other cognitive-behavioral components suggest that these strategies outperform usual care and non-CPT couples interventions in reducing physical and psychological IPV in military populations and high-income settings [92, 93]. Adapting and implementing these couples-based interventions will require additional formative research to ensure that there is not undue risk of integrating services for men into women’s protection and health programming in refugee settings.

In addition to the lessons learned regarding the intervention relevance, acceptability and feasibility, this study revealed opportunities to improve upon research procedures, including the mental health and violence assessment tools. First, questions about sexual violence displayed poor test-retest reliability. Discussion with interviewers and facilitators revealed that this was likely due to the sensitivity of these items, which led us to adding in a script to the assessment prior to administration of the sexual violence items restating that the participant’s responses were confidential. We also found poor internal consistency in the functional impairment measure, which led to a reduction in the number of items based on local stakeholder recommendations and item performance. Items that were removed were found to be irrelevant to indicators of functioning in the target population. For example, farming and income-generating activities are not common and, in some cases, forbidden in Nyarugusu camp and thus not representative of good daily functioning in this setting. Although the functional impairment measure was selected because it had been locally developed in a sample of women affected by gender-based violence in the eastern DRC, it is possible that the differences in context are extensive enough to necessitate developing, instead of adapting, a new measure of functioning through free listing and cross-cultural measurement development approaches [94].

### Strengths and limitations

This study possessed several strengths and limitations. First, to our knowledge this is the first study to document the development and adaptation of a multi-sectoral, integrated intervention to address mental health and IPV in refugees. The development and adaptation of the intervention was informed by a combination of qualitative and quantitative methodologies to provide insight into the contextual relevance, the acceptability of the intervention and related procedures, as well as the overall feasibility. This triangulation of methods and perspectives exposed opportunities to improve upon the intervention and research procedures prior to conducting a randomized feasibility trial.

In addition to these strengths, there are some key limitations that should be considered. First, many of the key adaptations were provided by our facilitators based on their impressions of the relevance, acceptability and feasibility of the intervention as well as comments made by the participants in the treatment cohort. It is possible that the recommendations shared by the facilitators reflect their opinions and, to a lesser degree, those of the participants. The exit interview was designed as an opportunity for participants to share feedback confidentially with research staff who were separate from the clinical facilitators; however, this opportunity occurred at the end of the intervention. Future adaptations to Nguvu should consider an iterative adaptation process employed throughout the intervention implementation process where participant’s opinions on the acceptability and relevance of the intervention are collected systematically. Retention in the intervention was another limitation that was reportedly due to a variety of barriers that were related to intervention procedures (e.g., communication problems) and others that were not related to the intervention (e.g., lack of childcare, competing priorities). Lastly, the treatment cohort participants were recruited from a single zone in the refugee camp. It is possible that there are differences between Congolese refugee women by zone, but there may also be implementation challenges that we did not experience, such as transportation and access, that could impact the feasibility of a study extended to women living in all zones in Nyarugusu refugee camp. Another limitation related to generalizability is that the participants represent women who have experienced IPV in the past year, which may exclude women with prior-to-past-year histories of IPV or other related histories of violence.

## Conclusion

In general we found that it was possible to implement an integrated health and protection intervention to address co-occurring psychological distress and IPV in a complex, dynamic refugee setting. Although feasible, we identified several challenges to implementation specific to the intervention content and delivery, administrative and research procedures, as well as systems-level factors, which may be informative in the design of similar multi-sectoral interventions for populations experiencing multiple adversities. The treatment cohort presented in this article provided insight into some unforeseen challenges that were addressed prior to the conduct of a larger feasibility randomized controlled trial. Forthcoming results from the randomized feasibility trial may provide indication as to whether the modifications improved the relevance, acceptability and feasibility of the intervention as well as whether it may be feasible to evaluate such a program through a large-scale, randomized controlled trial.

## Data Availability

The data generated from this study will be made available from the corresponding and senior authors on reasonable request.

## Abbreviations

AAS: Abuse Assessment Screen
CPT: Cognitive Processing Therapy
DRC: Democratic Republic of the Congo
HSCL-25: 25-Item Hopkins Symptom Checklist
HTQ: Harvard Trauma Questionnaire
ICC: Intraclass Correlation Coefficient
IPV: Intimate partner violence
PTEs: Potentially traumatic events
PTSD: Post-traumatic stress disorder
UNHCR: United Nations High Commissioner for Refugees
USA: United States of America
